# A CLINICOEPIDEMIOLOGICAL STUDY OF 50 CASES OF CUTANEOUS TUBERCULOSIS IN A TERTIARY CARE TEACHING HOSPITAL IN POKHARA, NEPAL

**DOI:** 10.4103/0019-5154.70670

**Published:** 2010

**Authors:** Binayak Chandra Dwari, Arnab Ghosh, Raju Paudel, P Kishore

**Affiliations:** *From the Department of Dermatology, Manipal Teaching Hospital & Manipal College of Medical Sciences Pokhara, Nepal*; 1 *From the Department of Pathology, Manipal Teaching Hospital & Manipal College of Medical Sciences Pokhara, Nepal*; 2 *From the Medicine, Manipal Teaching Hospital & Manipal College of Medical Sciences, Pokhara, Nepal*

**Keywords:** *Cutaneous TB*, *acid fast bacilli*, *anti-tubercular therapy*

## Abstract

**Background:**

Cutaneous tuberculosis (TB) is essentially an invasion of the skin by *Mycobacterium tuberculosis*, the same bacteria that causes pulmonary tuberculosis.

**Aim:**

This study was conducted to study the common types of cutaneous TB and to find the management pattern in a tertiary teaching hospital in Pokhara, Nepal.

**Materials and Methods:**

All the cases of cutaneous TB were biopsied and furthermore investigated by performing Mantoux test, sputum examination, fine needle aspiration cytology, chest X-ray and ELISA.

**Results:**

In this study, we found that tuberculosis verrucous cutis (48%) had a higher incidence than other types of cutaneous TB. More males were affected than were females (1.2:1). Commonly affected sites were the limb and the buttock (48%). The most commonly affected age group was 16–25 years (40%). All cases (except two) were more than 15 mm in size in the Mantoux test. The histopathological picture was typical in all except three cases. All patients were treated with antitubercular treatment as per the national guidelines.

**Conclusion:**

The most common type of cutaneous TB was tuberculosis verrucous cutis and the most commonly affected sites were the limb and the buttock. As cutaneous TB sometimes reflects the presence of pulmonary tuberculosis, its incidence should not be ignored.

## Introduction

Tuberculosis (TB) has been associated with humanity since ancient times. Robert Koch first discovered and isolated the tubercle bacillus, *Mycobacterium tuberculosis*, in 1882.[1] The invasion of skin by *M. tuberculosis* has become a rare event in developed countries. In the developing countries also, the incidence has fallen from 2 to 0.15%[2] and recently, it has fallen to 0.1%.[3,4] This may be due to antitubercular treatment (ATT), improved living standards, and BCG vaccination. Transmission is mainly by inhalation of airborne droplets and rarely by direct inoculation of the skin by *M. tuberculosis, M. bovis*, or the *Bacillus calmette-guerin* (BCG).[5] In 1956, Pillsbury, Shelly, and Kligman wrote, “in the skin tuberculosis presents itself in an astonishing variety of form”.[6] In 1981, Beyt *et al*. proposed a simplified scheme of classification which has gained wide acceptance.[7]

Exogenous

- Tubercular chancre
- Warty tuberculosis
- Lupus vulgaris

Endogenous

- Scrofuloderma
- Lupus vulgaris
- Tuberculous gumma

Tuberculides

- Lichen scrofulosum
- Papulo necrotic tuberculid
- Erythema nodosum
- Erythema induratum

Results of histological investigations are not characteristic of tuberculosis in the early stage, but tubercular granuloma does develop later with multinucleated giant cells and epithelioid cells. Caseous necrosis is also usually present in later stages.

A well-controlled clinical trial for cutaneous tuberculosis treatment is lacking and the results of trials for pulmonary tuberculosis treatment are often applied to cutaneous tuberculosis too. Clinical trials have confirmed the findings of animal experiments and have further shortened tuberculosis therapy to six months.[8,9]

## Materials and Methods

All the cases of cutaneous TB were identified in our Skin Outpatient Department over a period of 33 months from Sep 2005 to May 2008. The Mantoux test, sputum examination, fine needle aspiration cytology, chest X-ray, ELISA, and skin biopsy were done for diagnosis. Skin biopsies were sampled by 4 mm punch biopsy performed on the active advancing edge of the lesion under aseptic conditions. All the tissue samples were stained with hematoxylin and eosin as well as Ziehl Neelsen stain.

## Result

**Figure 1:**
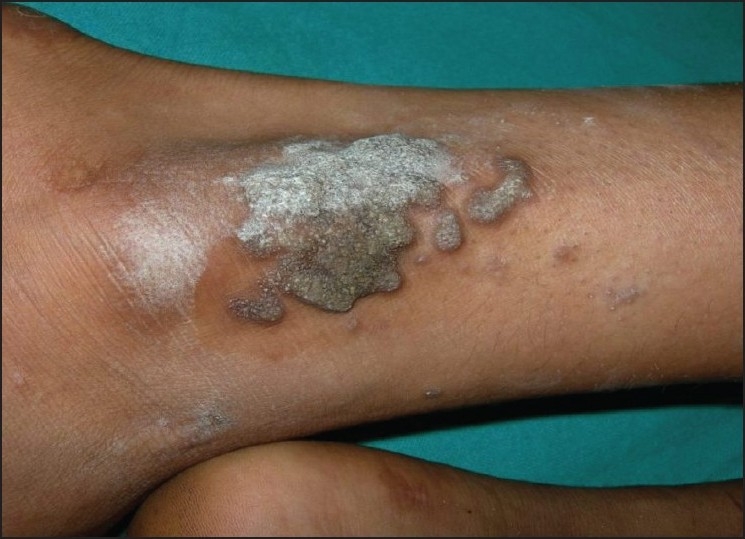
Tuberculois verrucoa cutis

**Figure 2:**
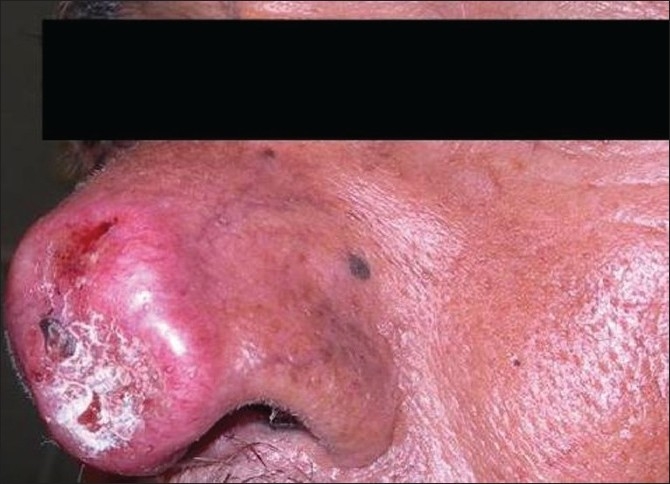
Lupus vulgaris

**Figure 3:**
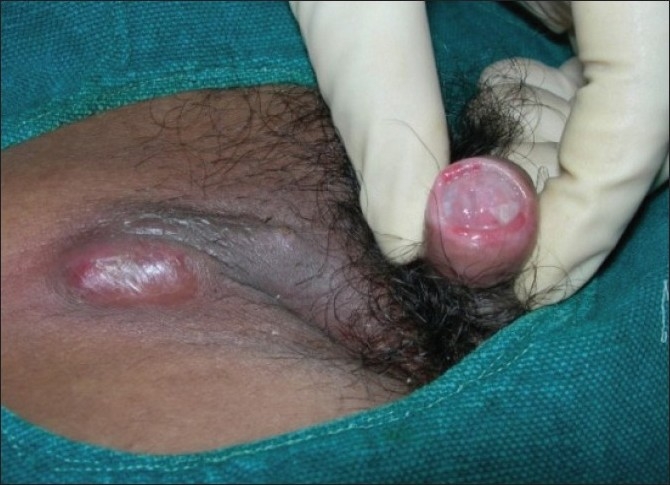
Scrofuloderma specify the lesions on glans

**Figure 4:**
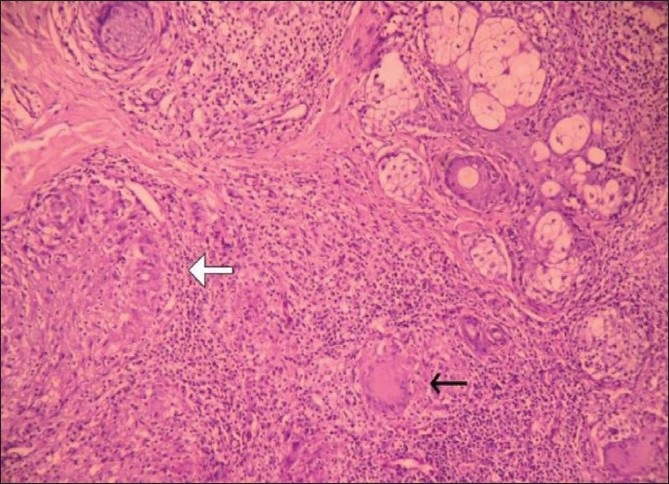
Epithelioid granuloma (white arrow) with Langhans giant cell (black arrow). Upper right corner shows pilosebaceous unit, (H and E stain, ×100)

**Figure 5:**
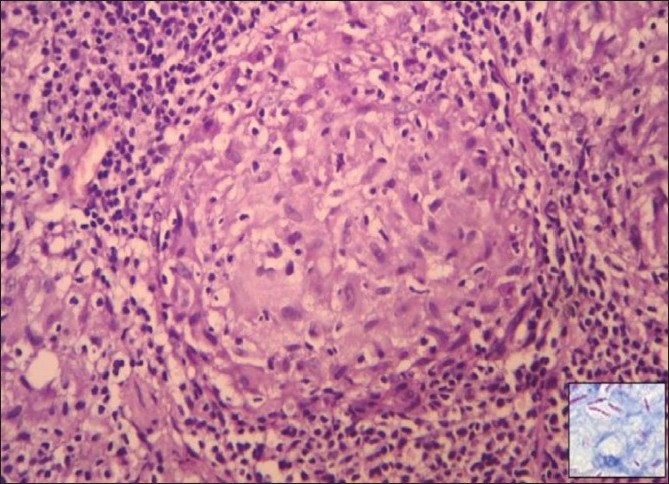
Epithelioid granuloma, (H and E stain, ×400). Inset shows acid-fast bacilli, (Ziehl Neelsen stain, ×1000)

**Figure 6:**
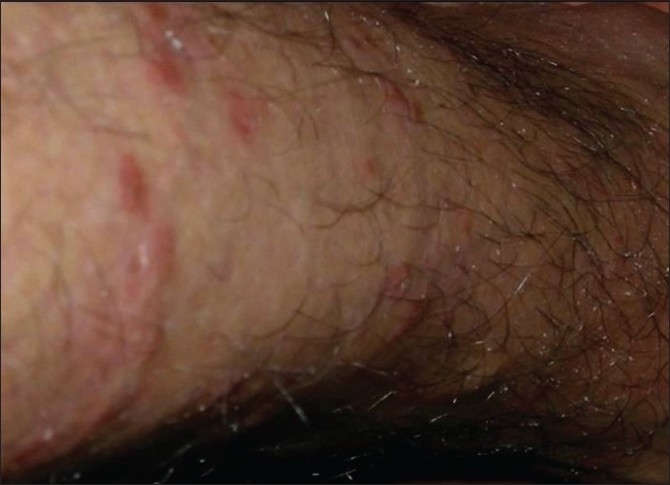
Erythema induratum

Fifty out of the 41000 (0.12%) patients examined in the OPD were included in the study. In this study, we found tuberculosis verrucoa cutis (TVC) (*n* = 24, 48%) was the most common type, followed by lupus vulgaris (LV) (*n* = 17, 34%) [Table 1, Figures 1 and 2]. We did not find any case of papulonecrotic tuberculosis in our study. Our youngest patient was nine years old and the oldest was 78 years old. Among the different age groups, the 16–25 years’ group was the most commonly affected group (*n* = 20, 40%) [Table 2]. All cases belonged to a low socioeconomic class. Most commonly affected sites were the limb and the buttock (*n* = 24, 48%) [Table 3] and males were more commonly affected than females (1.2:1). We also found coexistence of other diseases with cutaneous tuberculosis, of which diabetes mellitus (DM) was the most common (*n* = 7, 14%), followed by hypertension (*n* = 03, 6%), pulmonary tuberculosis (*n* = 2, 4%), and sporotrichosis (*n* = 2, 4%). One case each had squamous cell carcinoma (*n* = 1, 2%) and leprosy (*n* = 1, 2%) [Table 4]. The Mantoux test results were larger than 15 mm in size in all except two cases. Sputum test was positive for acid-fast bacilli in three cases and chest X-rays indicated pulmonary tuberculosis in two cases. Cervical lymphadenopathy were present in two cases and two cases had inguinal lymphadenopathy. Tissue exudates were aspirated only from scrofuloderma cases [Figure 3]. Out of seven cases, acid-fast bacilli (AFB) were seen in three cases and all were confirmed by Ziehl Neelsen stain. One patient was positive for HIV. Histopathological studies were done in all cases. Typical histopathological features of epithelioid granuloma [Figures 4 and 5], Langhans type of multinucleated giant cells, and caseous necrosis were seen in all cases except two patients in whom nonspecific chronic lymphohistiocytic inflammation was seen. Clinical findings of these two cases were like lupus vulgaris. Seventeen cases of TVC, three cases of LV, six cases of scrofuloderma, and none of erythema induratum [Figure 6] showed acid-fast bacilli in biopsy. All were treated with antituberculous treatment (ATT) and reviewed after two, four, six, eight, and ten months. All patients came to our outpatient department during followup, and all except two responded very well to eight months of ATT: first, an intensive therapy of isoniazide, rifampicin, and pyrazinamide for two months, and then, six months of isoniazide and ethambutol. The two patients who did not respond well to the eight months of ATT were treated with two months of isoniazide, rifampicin, ethambutol, and pyrazinamide followed by six months of isoniazide and ethambutol according to the National Tuberculosis Programme of Nepal. Patients were followed up for one year without any recurrence.

**Table 1:** Incidence and percentage of different clinical types

Clinical types	Case number (*n*) (total cases = 50)	Percentage of total cases
Tuberculois verrucoa cutis	24	48
Lupus vulgaris	17	34
Scrofuloderma	07	14
Erythema induratum	02	04

**Table 2:** Age distribution of the cases

Age group (years)	Case number (*n*) (total cases = 50)	Percentage of total cases
6–15	01	02
16–25	20	40
26–35	05	10
36–45	02	04
46–55	08	16
56–65	03	06
> 66	11	22

**Table 3:** Distribution of the involved sites

Anatomical site	Case number (*n*) (total cases = 50)	Percentage of total cases
Face	14	28
Neck	04	08
Trunk	08	16
Limb and Buttock	24	48

**Table 4:** Associated disorders

Co-existent diseases	Case number (*n*) (total cases = 50)	Percentage of total cases
Diabetes	07	14
Hypertension	03	06
Sporotrichosis	02	04
Pulmonary tuberculosis	02	04
Squamous cell carcinoma	01	02
Leprosy	01	02

## Discussion

Cutaneous TB is not uncommon, particularly in the developing countries. However, even in countries such as India and China where TB still occurs commonly, cutaneous outbreaks are rare (<1%).[5] In our studies, cutaneous TB was only about 0.12% of the total number of patients who visited the Dermatology Outpatient Department in the same period, much like the 0.1% incidence reported by Kumar.[3,4] Tuberculosis verrucous cutis was the most common type in our study and the study by Wong *et al*.[10] The most common site of involvement in our study was the limb and the buttock and inoculation TB was the commonest type. Wong *et al*. reported that the knee and the buttock was most common site in tuberculois verrucoa cutis,[10] similar to the findings of the limb being the most common site by the the study by Singh.[11] In our study, the 16–25 years’ age group was the most commonly affected, which was also noticed in the studies by Satyanarayan and Wong.[12,10] Males were found to be more commonly affected than females (1:2:1) like in other studies.[13,14] In our study, we reported some associated diseases like sporotrichosis, DM, hypertension, and pulmonary TB. Wong and Banerjee also noticed an association with pulmonary tuberculosis[10,15] while Restrepo noted an association with DM.[15] Lee *et al*. noticed tuberculous cellulite-like lesions in a patient who was diabetic and was taking an oral corticosteroid.[16] Decker *et al*. showed an association of HIV infection with tuberculosis.[17,18] Like Inamadar *et al*. and Pinto *et al*., we too noticed an association of cutaneous TB with leprosy in one case.[19,20] A few cases were associated with cervical lymhadenopathy and inguinal lymphadenopathy. In our study, ELISA for HIV gave negative results except for one case. We saw one case of squamous cell carcinoma which was also noticed by other authors.[21,22] The histopathological picture depends on the degree of the immune reaction and can be graded and organized along an immunopathological spectrum. The spectrum includes epithelioid granuloma with minimal necrosis and no acid-fast bacilli (AFB), indicating high immunity on one end, through necrotic epithelioid granuloma with some AFB, and to extensive necrosis with numerous AFB indicating low immunity on the other end. Clinically, the spectrum includes lupus vulgaris on the high immune end, through tuberculois verrucoa cutis towards scrofuloderma on the low immune end.[23] Other than the classical pattern of epithelioid granuloma, Langhans giant cells and caseous necrosis, several other patterns have been described and should be looked for.[23] These patterns include acute and chronic abscesses, diffuse infiltration of histiocytes, panniculitis, phlebitis, nonspecific chronic inflammation, naked nonnecrotic sarcoidal granuloma, and rheumatoid-like nodules.[24] In our studies, all the cases showed a classical picture except two cases which showed nonspecific chronic lymphohistiocytic inflammations. Similar histopathological features were seen in other studies also.[24] Santa Cruz and Strayer reported nonspecific chronic lymphohistiocytic inflammation in some cases as seen in the two cases in our study.[24] Like Lever, we also saw acid-fast bacilli in the biopsy report.[25] All except two cases were treated with ATT for two months with Isoniazide, Rifampicin, and Pyrazinamide, followed by six months with Isoniazide and Ethambutol which gave a very good response. But two cases who were associated with pulmonary tuberculosis, responded to Cat-1 treatment of ATT for the first two months with Isoniazide, Rifampicin, Ethambutol, and Pyrazinamide, followed by six months with Isoniazide and Ethambutol, according to the National Tuberculosis Programme of Nepal. All were followed up at an interval of two months with good results.

## Conclusion

Cutaneous TB is caused by *Mycobacterium tuberculosis*. The most common type was tuberculosis verrucous cutis and the most commonly affected sites were the limb and the buttock. Cutaneous TB may also be associated with diabetes and hypertension. The most commonly affected age group was the 16–25 years’ group and all our cases were treated with ATT. As cutaneous TB sometimes reflects the presence of pulmonary tuberculosis, its incidence should not be ignored.
